# Sunitinib in relapsed or refractory diffuse large B-cell lymphoma: a clinical and pharmacodynamic phase II multicenter study of the NCIC Clinical Trials Group

**DOI:** 10.3109/10428194.2011.555892

**Published:** 2011-04-04

**Authors:** Rena Buckstein, John Kuruvilla, Neil Chua, Christina Lee, David A Macdonald, Abdulwahab J Al-Tourah, Alison H Foo, Wendy Walsh, S Percy Ivy, Michael Crump, Elizabeth A Eisenhauer

**Affiliations:** 1 Sunnybrook Health Sciences Center, Toronto, ON, Canada; 2 Department of Medical Oncology and Hematology, Princess Margaret Hospital, University Health Network, Toronto, ON, Canada; 3 Cross Cancer Institute, Edmonton, AB, Canada; 4 QEII Health Sciences Centre, Halifax, NS, Canada; 5 BC Cancer Center-Fraser Valley Center, Surrey, BC, Canada; 6 NCIC Clinical Trials Group, Kingston, ON, Canada; 7 National Cancer Institute, Rockville, MD, USA; 8 Medical Oncology and Hematology, Princess Margaret Hospital, University Health Network, University of Toronto, Toronto, ON, Canada

**Keywords:** Sunitinib, large cell lymphoma, angiogenesis, CEC, CEP, biomarker

## Abstract

There are limited effective therapies for most patients with relapsed diffuse large B-cell lymphoma (DLBCL). We conducted a phase II trial of the multi-targeted vascular endothelial growth factor receptor (VEGFR) kinase inhibitor, sunitinib, 37.5 mg given orally once daily in adult patients with relapsed or refractory DLBCL. Of 19 enrolled patients, 17 eligible patients were evaluable for toxicity and 15 for response. No objective responses were seen and nine patients achieved stable disease (median duration 3.4 months). As a result, the study was closed at the end of the first stage. Grades 3—4 neutropenia and thrombocytopenia were observed in 29% and 35%, respectively. There was no relationship between change in circulating endothelial cell numbers (CECs) and bidimensional tumor burden over time. Despite some activity in solid tumors, sunitinib showed no evidence of response in relapsed/refractory DLBCL and had greater than expected hematologic toxicity.

## Introduction

Despite the improved cure rates achieved by adding rituximab to anthracycline-based chemotherapy in aggressive-histology B-cell lymphomas [1-4], relapsed or primary refractory diffuse large cell lymphomas continue to pose major clinical challenges, with disappointing results. Selected patients may be eligible for second-line therapy using high dose chemotherapy (HDCT) and autologous stem cell transplant (ASCT), but only 25-30% are cured [5]. In the remaining patients, palliative chemotherapy combined with corticosteroids offers temporary relief in some cases, but survival is typically short [6]. Therefore, there is an urgent need to identify alternative clinical approaches that either replace or enhance chemotherapy, offering better disease control with less toxicity.

Sunitinib maleate (SUTENT; Pfizer Inc., New York, NY), is an oral multi-targeted tyrosine kinase inhibitor of vascular endothelial growth factor (VEGF) receptors (VEGFR-1, -2, and -3) and platelet derived-growth factor receptors (PDGFR-α and -β) in addition to KIT, FLT3, RET, and CSF-1 [7,8]. This broad range of receptor inhibition may confer both antiangiogenic effects and direct anti-tumor effects, depending on the tumor subtype. Sunitinib 50 mg given on the schedule of 4 weeks on/ 2 weeks off provides progression-free and overall survival benefit in renal cell carcinoma (RCC) and progression-free survival benefit in imatinib-resistant gastrointestinal stromal tumors (GISTs) [8-10].

Given the previously identified angiogenic phenotype of large cell lymphomas [11-18], and the evidence that VEGF and PDGF may promote lymphoma cell growth in both a paracrine and an autocrine fashion [19-21], the NCIC (NCIC) Clinical Trials Group undertook a phase II study to evaluate the efficacy of sunitinib in patients with relapsed or refractory diffuse or mediastinal (thymic) large B-cell lymphoma (DLBCL and PMBCL) or transformed B-cell lymphomas. We chose a dose of 37.5 mg p.o. daily with no planned breaks, since this had demonstrated comparable benefit in GIST without an increase in toxicity [22], and the evidence from laboratory studies suggested that antiangiogenic agents have greater efficacy when given continuously without interruption [23,24].

## Materials and methods

### Patients

Adults aged 18 or older with relapsed or refractory DLBCL, PMBCL, or transformed lymphomas were eligible. Additional key inclusion criteria included at least one and no more than two prior cytotoxic chemotherapy regimens (one must have been anthracycline-containing). Salvage chemotherapy with HDCT/ASCT and up to one other chemotherapy and non-chemotherapy regimen (e.g. radiation) were permitted. Eligible patients must have been able to stop selected CYP3A4 inhibitors/inducers prior to starting sunitinib, have adequate cardiac function, have measurable bidimensional disease, and have an Eastern Cooperative Oncology Group (ECOG) performance status of 0-1. Key exclusion criteria were concurrent use of other antilymphoma therapy, prior use of sunitinib, other antiangiogenic agents, or multi-targeted receptor tyrosine kinase (RTK) inhibitors, uncontrolled hypertension, symptomatic cardio- or cerebrovascular disease, therapeutic anticoagulation, human immunodeficiency virus (HIV), and brain metastases. In addition, patients were excluded if they had a history of cerebrovascular accident, pulmonary embolism, or myocardial infarction within 12 months prior to study enrollment.

The study was approved by the institutional review boards of the participating NCIC Clinical Trials Group institutions and was registered with clinical-trials.gov. Written informed consent was obtained from all patients before study participation.

### Study design

This was a non-randomized, non-blinded multi-center phase II trial of sunitinib in patients with relapsed or refractory DLBCL or PMBCL conducted by the NCIC Clinical Trials Group. Sunitinib was supplied by the Cancer Therapy Evaluation Program (CTEP) of the US National Cancer Institute.

The primary endpoint of this study was objective response. Response was defined as per the report of the international workshop to standardize response criteria for non-Hodgkin lymphoma (NHL) [25]. The secondary endpoints included progression-free survival, toxicity, and the evaluation of antiangiogenic activity as determined by serial assessment of the number of circulating endothelial cells (CECs), apoptotic CECs (aCECs), and their precursors (CEPs).

### Treatment

Patients self-administered sunitinib 37.5 mg orally once daily in 4-week cycles. Dose modifications were made for toxicities graded according to the Cancer Therapy Evaluation Program, National Cancer Institute Common Terminology Criteria for Adverse Events (CTCAE) version 3.0. Up to two dose reductions (25 mg then 12.5 mg) were permitted for pre-specified toxicities. Grades 3-4 hematologic and grade 3 non-hematologic adverse events (AEs) generally required one dose reduction after resolution to <grade 2. Grade 4 non-hematologic AEs generally led to study discontinuation. Grade 2 hypertension was treated with antihypertensive medications until the blood pressure (BP) was controlled to a mild hypertension range. The drug was held for grade 3 hypertension until BP was controlled, then resumed with one dose reduction. Grade 4 hypertension led to study discontinuation. Patients requiring more than two dose reductions were removed from the study. No dose re-escalations were permitted. Patients who did not recover from toxic effects as required within 2 weeks were removed from protocol therapy.

### Assessments

Patients were clinically assessed every 4 weeks. Tumor imaging with computed tomography (CT) scans and assessment of cardiac function by electro-cardiography (ECG) and multi-gated acquisition (MUGA) scan were performed at baseline and every 8 weeks while the patient remained on study. Anatomic response assessments were performed locally at each site based on the largest bidimensional marker lesions identified at baseline. In the absence of serious or unmanageable toxicity, patients with complete response (CR), partial response (PR), or stable disease (SD) continued on therapy until disease progression or for a maximum of 12 cycles (1 year). Earlier discontinuation of therapy was permissible if continued treatment was no longer considered in the patient's best interest. In addition, patients who progressed (treatment failure) went off study at the time progression was documented clinically and/or radiographically. At the conclusion of the trial, a central review of X-rays and/or scans was to be carried out for any investigator-claimed responses.

CECs and CEPs were measured in three Ontario centers at baseline, day 1 of cycles 2 and 3, every 3 months thereafter, and at study discontinuation. Flow cytometric analysis was performed in one central location using previously published methods [26].

### Statistical methods

A Simon two-stage design was used [27]. A response rate of 5% was not considered promising, while a 20% response rate was worthy of further study. If no responses were seen in the first cohort of 15 evaluable patients, no further accrual would take place. If one or more responses were seen in group 1, then an additional 10 patients would be accrued. The study would be considered positive and sunitinib of interest in DLBCL and its variants if at least three responses were seen in the group of 25 patients (alpha: 0.12; beta: 0.89). All time-to-event data were described using the Kaplan-Meier method. Blood levels of CECs, aCECs, and CEPs (cells/mL) were plotted as percent change from baseline in comparison with the sum of bidimensional measurements of marker lymph nodes over time.

## Results

### Patient characteristics

A total of 19 patients were enrolled between February 2007 and September 2008 at seven Canadian sites. Two patients were deemed ineligible (one no histological diagnosis, one pulmonary embolism 5 12 months prior to entry). Seventeen patients were evaluable for toxicity and 15 were evaluable for response. Baseline patient characteristics are outlined in Table I. The median age was 65 and median time from lymphoma diagnosis was 20.3 months (range 5.8-132 months). Fourteen patients had a diagnosis of DLBCL, 10 had immediately preceding chemosensitive disease (complete or partial response to last treatment), and five had relapsed post-HDCT and -ASCT. The majority (11 patients) had an elevated serum lactate dehydrogenase (LDH) at the time of study enrollment.

**Table I:** Baseline characteristics.

Characteristic	No.
Median age, years (range)	65 (34–81)
Gender	
Female	7
Male	10
Performance status (ECOG)	
0	5
1	12
Prior chemotherapy	
1 prior chemotherapy regimen	7
2 prior chemotherapy regimens	10
High dose/ASCT	5
Rituximab	16
Prior radiotherapy	8
Best response to last chemotherapy	
Unknown	1
Complete response	7
Partial response	3
Stable disease	3
Progressive disease	2
Inevaluable	1
Number of sites of disease	
1	5
2	3
3	3
4 (or more)	6
Histology	
DLBCL	14
PMBCL	1
Transformed diffuse large B-cell	2
Baseline LDH	
≤ULN	6
>41–2.5 × ULN	4
>42.5–5 × ULN	3
>45 × ULN	4

ECOG, Eastern Cooperative Oncology Group; ASCT, autologous stem cell transplant; DLBCL, diffuse large B-cell lymphoma; PMBCL, primary mediastinal B-cell lymphoma; LDH, lactate dehydrogenase; ULN, upper limit of normal.

### Treatment delivery

The median number of cycles of sunitinib received was 2 (1-5), with only five patients remaining on drug for three or more cycles. Only six of 17 patients received >90% of the planned dose intensity, with 14 patients missing doses and five undergoing dose reductions necessitated by toxicities (Tables II and III).

**Table II:** Most common adverse events according to grade.

	Grades 1–2		Grades 3–4		Total	
Adverse event	No.	%	No.	%	No.	%
Hypertension	1	6	3	18	4	24
Fatigue	7	41	3	18	10	59
Anorexia	6	35	2	12	8	47
Dehydration	3	18	–	–	3	18
Diarrhea	4	24	2	12	6	35
Heartburn	4	24	–	–	4	24
Mucositis (clinical exam)	4	24	–	–	4	24
Mucositis (functional/symptomatic)	3	18	–	–	3	18
Nausea	8	47	–	–	8	47
Taste alteration	3	18	–	–	3	18
Vomiting	5	29	–	–	5	29
Pain oral cavity	3	18	–	–	3	18

**Table III:** Hematological adverse events.

	Grade				
Adverse event	0	1	2	3	4
Granulocytes	4	2	6	5	–
Hemoglobin	1	8	5	2	1
Lymphopenia	3	2	6	4	2
Platelets	3	7	1	3	3
Leukocytes	4	2	4	7	–

### Safety

The most commonly reported non-hematologic treatment-related AEs thought to be at least possibly related to sunitinib were: fatigue (59%), anorexia (47%), nausea (47%), diarrhea (35%), vomiting (29%), mucositis, clinical exam and functional/ symptomatic (24% and 18%, respectively), heartburn (24%), and hypertension (24%), with most of these events of mild or moderate intensity (grades 1 or 2) (Table II). One patient had a grade 2 asymptomatic reduction in left ventricular (LV) systolic function, two developed grade 1 pleural effusion, and four developed elevated thyroid stimulating hormone (TSH) on treatment, although only two required thyroid replacement. One patient had a grade 4 pericardial effusion develop on study, but this was deemed to be related to progressive lymphoma, not to sunitinib. Neutropenia and thrombocytopenia were grade 3 or more in five and six patients, respectively (Table III), and were the most common reason for dose omission or reduction. Six patients (35%) discontinued treatment due to AEs, four of which were hematologic, and eight patients discontinued therapy due to disease progression. There were no treatment-related deaths.

### Efficacy

**Figure 1:**
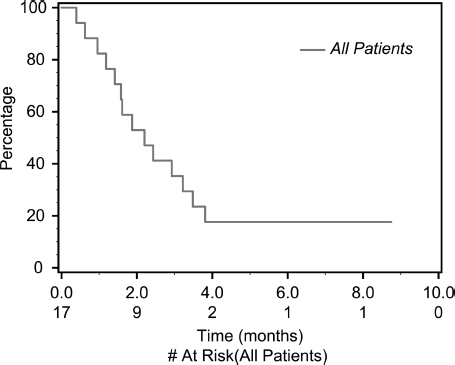
Overall survival.

Of 17 eligible patients, 15 were evaluable for response. One patient received only two doses of drug, and one patient did not have restaging scans. Of those evaluable, no patient experienced a clinical response to sunitinib after central radiology review. As a result, the study was closed to accrual according to the protocol. Nine patients (53%) achieved stable disease as best response (median duration 3.4 months; range: 1.4-8.7 months), and six (35%) had primary progressive disease. Overall progression-free survival (PFS) (Figure 1) was 2.2 months (95% confidence interval [CI] 1.41-3.48). All patients are currently off study, eight due to disease progression, one due to symptomatic progression, and six due to toxicity, and two withdrew consent.

### Analysis of biomarkers

**Figure 2:**
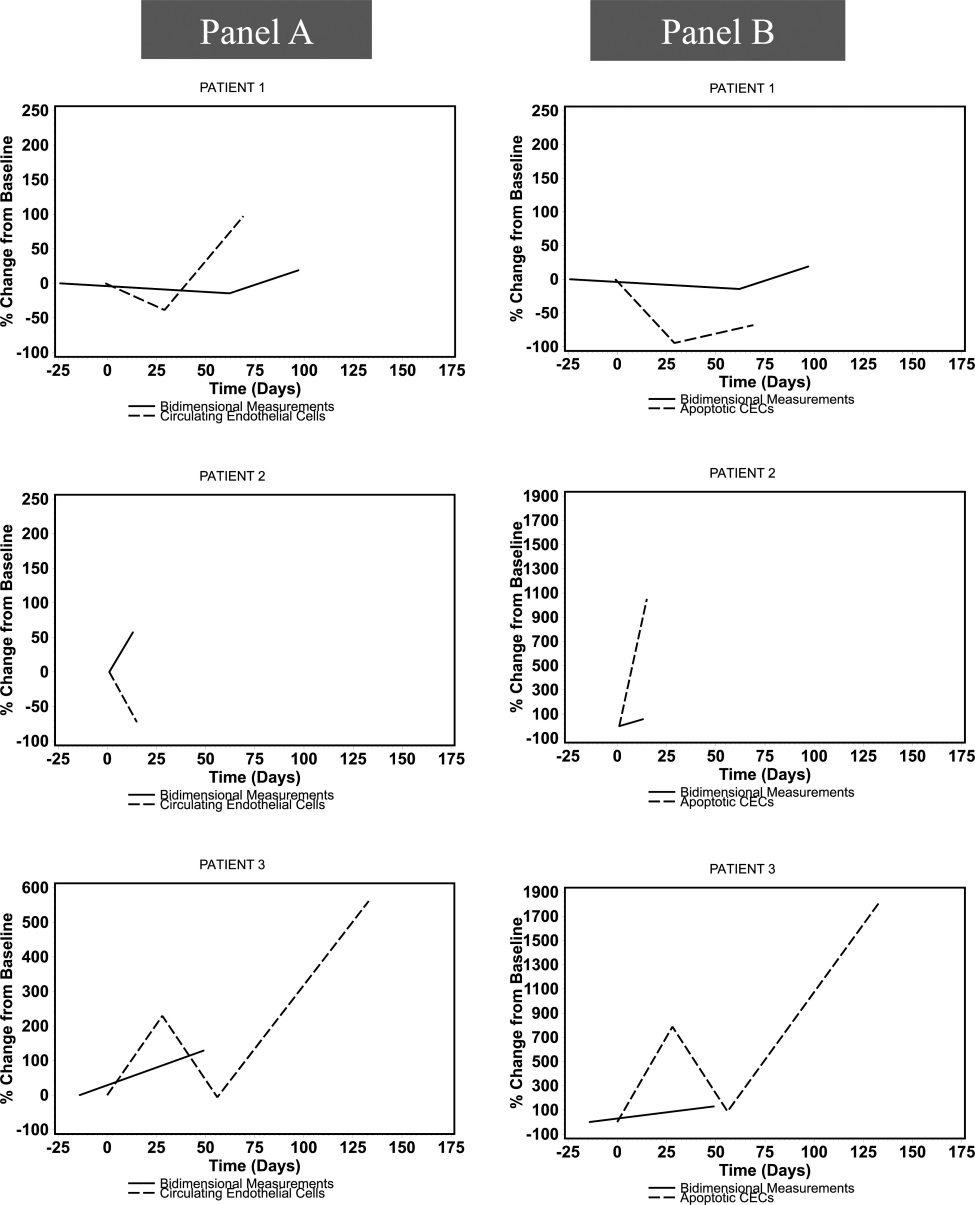

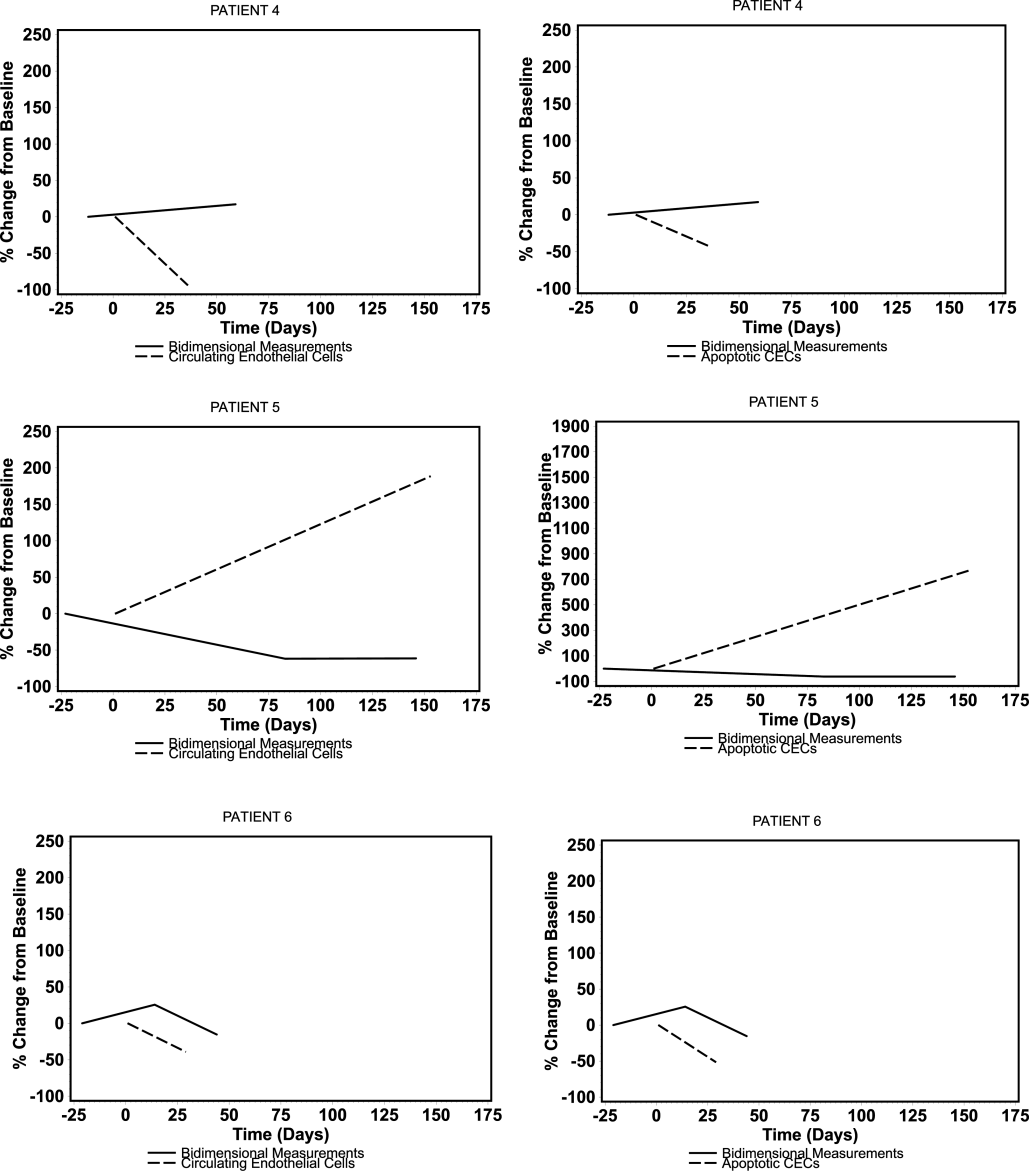
Percent change (from baseline) in bidimensional measurements compared with percent change in circulating endothelial cells (panel A), and apoptotic circulating endothelial cells (panel B) over time measured in days. CECs, circulating endothelial cells.

CECs, aCECs, and CEPs were assessed at baseline in 10 patients and in two or more serial measurements in seven patients (six of whom had restaging CT scans for comparison). The median baseline CEC count was 2.9 cells/mL (range 1.13-7.03 cells/ mL), of which 86% (range 30-99%) were viable. CEP levels were too low to be serially followed. There was no discernible relationship between the change in absolute or apoptotic CECs over time and clinical response or change in bidimensional measurements (Figure 2). Sixty-seven percent of the patients with stable disease had a normal LDH at baseline compared with 0% in patients with primary progressive disease.

## Discussion

As in solid tumors, neo-angiogenesis may contribute to the pathogenesis and poor prognosis in many aggressive-histology lymphomas. The detection of VEGF A, B, and C isoforms and their receptors on many large cell lymphoma samples suggests that the VEGF pathway is critically important, and may contribute to disease progression in both an auto-crine and a paracrine fashion [12,17,18,28,29].

The anti-VEGF monoclonal antibody bevacizu-mab has been evaluated in relapsed DLBCL, resulting in one partial response and eight patients with stable disease as best response, out of 30 evaluable patients; 6-month PFS, the primary study endpoint, was 15% (95% CI 5-26%) [30].

Unfortunately, a multicenter phase III trial comparing CHOP-R (cyclophosphamide, doxorubicin, vin-cristine, prednisone, and rituximab) with CHOP-R + bevacizumab was recently discontinued due to excess cardiac morbidity, and the clinical benefits of adding an antiangiogenic agent to standard treatment are still unknown.

The evaluation of agents targeting VEGF signaling in NHL, notably DLBCL, is of interest. Sunitinib (SU11248) was a logical agent to study, since it is an orally bioavailable inhibitor affecting RTKs involved in tumor proliferation and angio-genesis, including VEGFR-1, -2, and -3, and PDGFR-α and -β.

In this multicenter phase II study, sunitinib 37.5 mg p.o. daily did not produce objective radi-ologic responses in patients with relapsed or refractory DLBCL or transformed lymphoma, with shortlived stable disease as the best achievable response in 53% of patients. This contrasts with objective response rates of 25.5-36.5% in metastatic RCC [10], 23% in bevacizumab-refractory metastatic RCC [31], 16% in advanced pancreatic neuroendo-crine tumors [32], and 11% in advanced non-small cell lung cancer [33]. The lack of response in lymphoma is congruent with the sunitininb experience in heavily pretreated chronic lymphocytic leukemia (CLL) [34].

These negative results may be explained by a number of factors. First, diffuse large cell lymphomas are rapidly proliferating tumors that may not be suited to treatment with cytostatic agents used as monotherapy. The median time on drug (2 months) may have been too short to demonstrate any efficacy in many patients whose baseline elevated LDH suggested highly mitotic tumors. Second, the dose of sunitinib chosen for testing in this patient population, 37.5 mg daily, may have been too low, despite the continuous schedule used. Most clinical trials of sunitinib reporting significant objective response rates have used 50 mg daily for 4 out of 6 weeks. We selected the lower dose to permit continuous administration and to avoid the rebound increase in markers of angiogenesis observed after angiogenesis inhibitors are stopped [35-37]. However, despite a lower daily dose, we encountered unexpected excessive myelosuppression induced by sunitinib, which compromised the ability to administer even the reduced dosage intended in this study on schedule, and accounted for many of the AEs that led to study treatment discontinuation. This may be because 37.5 mg daily is a comparable if not slightly higher total dose over a 6-week period than the total dose of the 50 mg syncopated 6-week schedule. Or perhaps, in this patient population who have been previously treated with multiagent chemotherapy (including alkylators and anthracyclines) and, frequently, ASCT, an interrupted schedule (as has been evaluated in patients with solid tumors) may have allowed greater drug delivery. Our hematologic adverse event experience is not dissimilar to that reported in heavily pretreated patients with CLL given sunitinib 37.5 mg p.o. daily [34]. In that trial, 16 of 18 (89%) patients experienced grade 3 or higher adverse events to sunitinib, with 56% ≥grade 3 thrombocytopenia and 27% ≥grade 3 neutropenia. These data, together with those from our trial, suggest that patients with lymphoid cancers may experience greater myelosuppression because of the presence of extensive marrow involvement, as might be expected in patients with CLL, or due to the nature of their prior therapy, such as exposure to purine analogs or following ASCT.

Clinical trials for traditional cytotoxic drugs are often designed to show an improvement in the objective response rate. Many of the newer anti-cancer agents, including those targeting angiogenesis, may have a primarily cytostatic rather than a cytotoxic effect, and may delay progression and/or death while having little effect on tumor size. In the absence of randomized trials wherein time-to-event endpoints such as time to tumor progression can be reliably compared with a control group [8,10], surrogate biomarkers are needed to help validate the mechanistic hypotheses of action, identify responsive patients and optimal biologic doses, and predict the outcomes of regimens that include anti-VEGF agents.

CECs and their progenitors (CEPs) are rarely found in the blood of healthy subjects, but may be elevated in patients with neoplastic disease and correlate with angiogenesis [38]. Preclinically, CECs appear to correlate with tumor volumes in SCID (severe combined immunodeficiency) mice bearing human lymphoma [39]. Increased CD133+CD34+VEGFR-2+ endothelial precursor cells (EPCs) are detectable in the peripheral blood of patients with aggressive lymphomas and decrease in number following complete response to chemotherapy [40]. Additionally, bevacizumab reduced the frequency of viable CECs and CEPs in patients with rectal cancer [41], and in a previous study in patients with relapsed aggressive lymphomas, CECs and CEPs declined during metronomic low-dose cyclo-phosphamide and high dose celecoxib [42].

The lack of correlation between tumor response as measured by bidimensional measurements and changes in CECs contrasts with the observations in patients with RCC treated with sunitinib on a 50 mg daily for 4 weeks, with 2 weeks off, schedule [43]. While on sunitinib, opposite kinetics of two circulating CD34^bright^ cell populations, hematopoietic progenitor cells (HPCs) and small CECs, were observed, with the HPCs decreasing and the CECs increasing but normalizing to pretreatment values during the 2-week drug-free period. This suggested that sunitinib was directly targeting the immature tumor vessels. In another study, sunitinib was reported to cause a greater increase in CECs in patients with GIST, and this increase was associated with clinical benefits compared with patients with progressive disease [44].

The problematic reproducibility and validity of measuring low frequency CECs by flow cytometry is known, but our negative findings may simply reflect the limited power of this analysis due to serial monitoring of a small number of patients (*n* = 8) for a median of two cycles.

Finally, cytostatic agents probably work best when used in combination with chemotherapy. Despite this, it would be unrealistic to pursue more drug development with sunitinib in combination with chemotherapy in the absence of single-agent activity (or tolerability). The absence of any objective responses negatively predicts for eventual regulatory approval of a given therapeutic agent in solid tumors. There is no reason to suppose that in lymphomas, which are often more sensitive to a specific che-motherapeutic agent than are solid tumors, this observation with respect to targeted agents would not also hold true. Indeed, all recently approved new agents in lymphoma demonstrated objective responses when given as single agents [45].

## Conclusion

Sunitinib administered 37.5 mg p.o. daily was inactive in patients with relapsed or refractory DLBCLs and resulted in greater hematological and other toxicities compared to the experience in populations with solid tumors. No convincing pharmacodynamic evidence of antiangiogenic activity was demonstrable by CEC and CEP biomarker analysis, with the qualification that limited serial sampling was possible.

In our opinion, this study illustrates the challenge of studying novel targeted therapies including antiangio-genesis agents in rapidly proliferating lymphomas.
